# Editor’s introduction

**DOI:** 10.1186/s41039-015-0015-z

**Published:** 2015-07-15

**Authors:** Siu Cheung Kong

**Affiliations:** Department of Mathematics and Information Technology, The Hong Kong Institute of Education, 10 Lo Ping Road, Tai Po, New Territories Hong Kong

The current issue of *Research and Practice in Technology Enhanced Learning* is delighted to present a Special Issue on the theme “Emerging Trends for Open Access Learning” with three selected articles—on top of our regular publication of original articles.

The Special Issue on the theme “Emerging Trends for Open Access Learning,” with Rachid Benlamri and Fanny Klett as Guest Editors, aims to disseminate advanced technological solutions as well as novel methodical approaches for open access to learning and education. The Editorial by the Guest Editors delineates the theme of this Special Issue as well as introduces the individual articles. I wish to express my sincere gratitude to the Guest Editors for their great effort and professional contribution to the realization of the Special Issue.

In addition to the three Special Issue articles, this issue presents three original articles looking into a multi-layer map model for Web-based learning, students’ interaction with the tutorial dialogs in an intelligent tutoring system (ITS), and an active learning strategy with visualization in instructor-mediated classrooms.

In the paper *A Multi-layer Map-oriented Resource Organization System for Web-based Self-directed Learning Combined with Community-based Learning*, Li, Hasegawa, and Kashihara propose a multi-layer map model that visualizes learners’ basic learning behaviors when they learn under the methodology of topic maps. Based on a case study, the authors confirm the potential of the proposed model to support learners to better engage in self-directed learning with tasks on searching and organizing resources from the Web to create a knowledge structure. The authors also discuss further directions on improving the designed model and strengthening its use for community-based learning.

In the paper *Investigating Student Interactions with Tutorial Dialogues in EER-Tutor*, Elmadani et al. investigate how students interact with the tutorial dialogs in an ITS entitled EER-Tutor, which analyzes both eye gaze data and student-system interaction logs to detect students’ sub-optimal behaviors and make corresponding real-time adaptation in learning environments. The authors identify the differences between novices and advanced students in interacting with the tutorial dialogs in an ITS, and discuss the possibility to predict future errors based on the tutorial dialogs that a student has received.

In the paper *Effect of Active Learning Using Program Visualization in Technology Constrained College Classrooms*, Banerjee, Murthy, and Iyer explore the feasibility of the implementation of active learning strategy with visualization in instructor-mediated classrooms. The authors compare how two different engagement levels with visualization, namely “Viewing” and “Responding,” among students impact their behavioral engagement, affect and perception of learning, as well as cognitive achievement. The authors also discuss the educational potential of the designed instructional strategy.

We keep soliciting an eclectic collection of quality paper submissions from researchers and practitioners around the world to share insights into the theoretical and methodological dimensions of research and practice in technology-enhanced learning.

Kong, S. C.

Editor-in-Chief

